# Impact of Possession and Player Position on Physical and Technical-Tactical Performance Indicators in the Chinese Football Super League

**DOI:** 10.3389/fpsyg.2021.722200

**Published:** 2021-09-29

**Authors:** Tianbiao Liu, Lang Yang, Huimin Chen, Antonio García-de-Alcaraz

**Affiliations:** ^1^School of Physical Education and Sports Science, Beijing Normal University, Beijing, China; ^2^Department of Education, Faculty of Education Sciences, University of Almería, Almería, Spain; ^3^SPORT Research Group (CTS-1024), University of Almería, Almería, Spain

**Keywords:** team sports, performance analysis, game demands, player role, physical performance, technical indicators

## Abstract

The purpose of this study was to investigate the impact of high (HPBPT) and low percentage ball possession teams (LPBPT) on physical and technical-tactical performance indicators in the Chinese Football Super League (CSL). Eight physical performance indicators and 26 technical-tactical performance indicators from all 240 matches from season 2018 were analyzed, as well as three contextual variables (team strength, quality of opposition, and match location). Players were divided according to five positions: fullbacks, central defenders, wide midfielders, central midfielders, and attackers. A k-means cluster analysis was conducted to classify all match observations into two groups: HPBPT (*n* = 229) and LPBPT (*n* = 251). A mixed linear model was fitted with contextual variables as covariates. When significant interactions or main effects were detected, a *post hoc* comparison was used to compare physical and technical/tactical differences between HPBPT and LPBPT. Results showed that central defenders and fullbacks covered more high-intensity and sprint running distance in the high possession teams, while wide midfielders and forward covered more high-intensity and sprint running distance in the low possession teams. Meanwhile, players from high ball possession teams were strong in technical indicators, especially in attacking organization. These results may help coaches to understand current football development trends and develop suitable training plans and tests for elite football players.

## Introduction

Possession in football is a basic and important performance indicator (Pollard and Reep, 1997). Currently, ball possession is a trending topic that is heatedly discussed because of the success of possession-play teams in the World Cup and European Champions league. In the English Premier League, it was found that the best and most successful teams record longer possessions (Jones et al., 2004; Bloomfield et al., 2005; Carling et al., 2005). Additionally, in La Liga (Spanish League), possession was found to be a strong indicator in predicting the winning team (Lago-Peñas and Dellal, 2010; Lago-Peñas et al., 2010), and in the Chinese Super League (CSL), higher ranked teams were associated with high ball possession (Liu et al., 2019).

Possession is very much related to ball control and playing style. There are traditionally two typical playing styles that are most commonly described: (a) direct play and (b) possession play (Bate, 1988; Hughes and Franks, 2005; Lago-Peñas and Dellal, 2010; Kempe et al., 2014). A direct playing team may have less possession on the pitch, and their players tend to play more in counterattack (Tenga et al., 2010a,b; Tenga and Sigmundstad, 2011; Fernandez-Navarro et al., 2016). In contrast, a possession playing team will keep the ball for a long time (Kempe et al., 2014), and their players tend to want the ball close to the goal to minimize giving ball control to their opponent. Common beliefs are that Spanish football styles involve possession play and English styles involve more direct play (Sarmento et al., 2013), but recent studies have found that mixed play strategies also work during the match Jones et al. (2004). Different playing styles are rooted in different football philosophies, and each playing style has led to great achievements in history and is still being discussed today (Hughes and Franks, 2005; Sarmento et al., 2013; Yi et al., 2019). Bloomfield et al. (2005) found in three elite English clubs that “all were possession dominant, some already absorbed pressure and adopted a counterattacking strategy.” In addition, in 2018, World Cup teams that entered the top 16 mostly used mixed playing styles (Yi et al., 2019).

Since high and low ball possession typically represent different playing styles that are related to different playing variables (Hewitt et al., 2016), previous researchers have found that high or low ball possession had an impact on technical-tactical and physical performance indicators (Bradley et al., 2013b; da Mota et al., 2016). Technically, high possession teams tend to have longer ball control and more passes (Tenga et al., 2010a), while low possession teams have less ball control and fewer passes. It was expected that physical performance indicators would be affected by high and low possession because playing against high-quality opponents has been linked to lower ball possession (Bloomfield et al., 2005; Lago, 2009), and it is possible for such teams to run more at high intensity to regain the ball (Di Salvo et al., 2009; Bradley et al., 2013b). However, there is also evidence that suggests the opposite is true (Bradley et al., 2013b; da Mota et al., 2016).

In addition to the effects of ball possession, player role was found to be another important variable. Performance indicators are different for different playing positions. Several studies have already focused on technical-tactical and physical profiles for playing positions (Di Salvo et al., 2009; Dellal et al., 2011; Liu et al., 2016). From a physical point of view, wide midfielders were reported to cover the greatest distances at high intensity compared to other positions (Bradley et al., 2009; Di Salvo et al., 2009; Mallo et al., 2015). Moreover, technically, center midfielders made more passes than forward and central defenders (Taylor et al., 2004; Redwood-Brown et al., 2012). However, separate analyses for technical-tactical and physical indicators are not adequate because team performance in a real match is affected by interacting performance indicators, which warrant more complex studies.

The exploration of the relationship between playing style (related to ball possession) and player performance, also linked to player role (Fernandez-Navarro et al., 2016; Yi et al., 2019), is still an important and suitable indicator that is popularly used to evaluate performance (Bradley et al., 2013b; da Mota et al., 2016) and identify the best teams at the top level (Lago, 2009; Yang et al., 2018). Although there was an early study that did not support possession play (Bate, 1988), it still stated the importance of entering attacking third zones and therefore creating a chance of scoring (Bate, 1988). Indeed, there is evidence that high possession teams have a greater chance of attacking third and opposition penalty areas (Tenga et al., 2010a; da Mota et al., 2016). Moreover, contextual variables like team strength, quality of opposition and match location are also important (Lago-Peñas and Dellal, 2010; Bradley et al., 2014; Liu et al., 2021) because they also influence the team’s playing style (Fernandez-Navarro et al., 2018) and players’ performance. Currently, most studies are centered on European leagues and the World Cup, while there is still a knowledge gap with respect to the CSL. This football league is growing quickly and its performance evolution has progressed rapidly (Zhou et al., 2020). Although the CSL has received considerable attention in the last few years (Gai et al., 2018; Yang et al., 2018; Zhou et al., 2018; Liu et al., 2019), most studies are focusing on an analysis of performance indicators with a lack of discussion on CSL teams’ playing styles. Considering the great evolution of football play in this decade and ball possession as a representative indicator of the playing style, this study aims to analyze the impact of ball possession on physical and technical-tactical indicators in terms of playing position in the CSL.

## Materials and Methods

### Samples and Variables

Data were collected from all the matches (*n* = 240) played in the CSL during the 2018 season. A total of 16 teams participated in this competition, playing 30 matches each in a balanced home and away schedule (15 home and 15 away matches). Players were divided according to five positions as in previous studies (Bush et al., 2015a,b): fullbacks (*n* = 1,120), central defenders (*n* = 1,072), wide midfielders (*n* = 1,404), central midfielders (*n* = 1,669), and attackers (*n* = 898). Match data on goalkeepers were excluded because of the specificity of this position; hence, 6,163 match observations were included. Based on previous studies (Bradley et al., 2009; Di Salvo et al., 2009; Liu et al., 2016, 2019, 2021; Gai et al., 2018; Zhou et al., 2018, 2020; Gong et al., 2021), eight physical performance indicators and 26 technical-tactical performance indicators as well as three contextual variables (team strength - TS-, quality of opposition -QO- and match location -ML-) were analyzed (Table 1). Player position and ball possession were also measured. This study was conducted according to the ethical principles of the World Medical Association Declaration of Helsinki (World Medical Association, 2013).

**TABLE 1:** Match performance indicators in Chinese Super League.

**Physical performance-related parameters**	
Total distance (m)	Distance covered in the match
Sprint distance (m)	Distance covered at a speed over 23 km/h
Sprint distance in possession (m)	Sprinting distance covered in ball possession
Sprint distance out of possession (m)	Sprinting distance covered when the opponent has the ball possession
High-intensity distance (m)	Distance covered at a speed of 19.1–22.9 km/h
High-intensity distance (in possession (m)	High-speed running distance covered in ball possession
High-intensity distance out of possession (m)	High-speed running distance covered when the opponent has the ball possession
High-intensity interval (s)	Average time between high-intensity runs
**Technical performance-related parameters**	
Shots	An attempt to score a goal, made with any (legal) part of the body, either on or off target
Shots on target	An attempt to score a goal which required intervention to stop the ball going in, or resulted in a shot that would have gone in without diversion
Shots success rate (%)	Shots on target as a proportion of total shots
Possession rate (%)	The duration from a player taking over the ball as a proportion of total duration when the ball was in play
Possession in opponent’s court (%)	Possession by a team in the opponent’s half of the pitch
Challenges won (%)	Challenge duels won by a team as a proportion of total duels of the match
Total passes	A ball sent from one player to another
Successful passes	An intentionally played ball sent from one player to another that receives the ball
Pass success rate (%)	Successful passes as a proportion of the total passes
Forward passes	An intentional played ball sent from one player to another who is located closer to the opponent goal
Success rate of forward pass (%)	Successful forward passes as a proportion of the total forward passes
Opponent 35-m entry	Number of times when the ball (possessed by the attacking team) enters the 35 m area of the opponent’s half of the pitch
Opponent penalty area entry	Any ball sent into the opposition team’s area from a wide position
Aerial challenges	Aerial duels
Number of successful aerial challenges	Successful aerial duels
Success rate of aerial challenges (%)	Aerial duels won by a team as a proportion of total duels performed in the match
Ground challenges	Ground duels
Number of successful ground challenges	Successful ground duels
Success rate of ground challenges (%)	Ground duels won by a team as a proportion of total duels of the match
Crosses	Any ball sent into the opposition team’s area from a wide position
Crosses success rate (%)	Successful crosses as a proportion of total crosses
Corners	Ball goes out of play for a corner kick
Offside	Being caught in an offside position resulting in a free kick to the opposing team
Foul	Any infringement that is penalized as a foul by a referee
Yellow card	Referee decision for reasons of foul play, persistent infringement, hand ball, dangerous play, time wasting, etc.
Red card	Referee decision for reasons of foul play, serious foul, violent behavior, using offensive, insulting or swear words, showing a yellow card for the second time, etc.
**Contextual parameters**	
Match Location (ML)	Venue of the match—playing at home or away
Team Strength (TS)	Competitive level of the competing team according to the end-of-season points
Quality of Oppositions (QO)	Competitive level of the opposing team according to the end-of-season points

### Procedure

Team performance data were originated from Amisco^®^ Sports Analysis Services, and these data were ordered in specific spreadsheets. During the observation procedure, the movements of all field players in each match were tracked by eight stable and synchronized cameras positioned at the top of the stadium at a sampling frequency of 25 measures a second. Signals and angles obtained by the encoders were sequentially converted into digital data and recorded on six computers for post-match analyses. From the stored data, the distance covered, time spent in the different movement categories, and the frequency of occurrence for each activity were determined by Athletic Mode Amisco^®^ Pro, Nice, France (Di Salvo et al., 2007). The reliability of this data source and collection methods were previously validated and used by many studies (Lago-Peñas et al., 2009; Zubillaga et al., 2009; Dellal et al., 2011; Castellano et al., 2014; Valter et al., 2017).

### Statistical Analysis

Descriptive statistics (mean ± SD), were calculated for each variable. To establish whether a team was in high or low ball possession during a match, a cluster *K*-means analysis (Schwartz’s Bayesian) was taken based on the team’s ball possession in every match across the whole season (Bradley et al., 2013b; da Mota et al., 2016). The average ball possession in a high ball possession team (HPBPT) was 56.53 ± 4.22% (range of 51–69%; *n* (229), and in a low ball possession team (LPBPT), was 43.98% (±4.44% (range of 31–%-50%; *n* = 251).

Finally, a mixed linear model was fitted in which team strength (TS), Quality of Opposition (QO), and Match Location (ML) were used as covariates. When significant interactions or main effects were detected, a *post hoc* comparison was used to compare physical and technical/tactical differences between HPBPT and LPBPT. All analyses were conducted using lmerTest (Kuznetsova et al., 2017) and emmeans (Russell, 2021) packages in statistical software R (ver. 4.1.1) (R Core Team, 2021) with significance set at *p* < 0.05.

## Results

### Physical Performance

The physical performances of HPBPT and LPBPT are shown in Table 2. The high-intensity distance (first half and total) in ball possession were higher in HPBPT than in LPBPT (*p* < 0.05). When the ball was out of possession, high-intensity running (first half and total) of HPBPT was lower than that of LPBPT (*p* < 0.05). No other differences were observed for other running indicators between HPBPT and LPBPT. As covariates, TS had a major influence on high speed running in possession, QO had a major influence on high speed running out of possession, and ML influenced high speed performance both in and out of possession.

**TABLE 2:** Difference of running performance between HPBPT and LPBPT.

Performance indicators	HPBPT	LPBPT	df	Statistic(t)	*p*-value	*Post hoc* comparison	Team strength		Quality of opposition		Match location	
									
							Statistic(t)	*p*-value	Statistic(t)	*p*-value	Statistic(t)	*p*-value
Total distance 1st half	52,816.07 ± 2468.98	52,928.18 ± 2571.41	474.375	1.244	0.214		1.771	0.204	6.327	0.012	0.820	0.366
Total distance 2nd half	53,047.47 ± 2944.94	52,885.12 ± 3164.31	473.824	–0.068	0.946		0.429	0.523	2.049	0.153	0.305	0.581
Total distance	105,856.63 ± 4573.39	105,769.16 ± 4882.77	474.961	0.608	0.543		1.182	0.295	4.506	0.034	0.851	0.357
High-intensity distance 1st half (in possession)	1,218.14 ± 331.49	1,080.86 ± 311.03	474.719	–2.520	0.012	H > L	12.624	0.003	0.964	0.327	16.046	<0.001
High-intensity distance 2nd half (in possession)	1,227.00 ± 319.17	1,130.39 ± 338.32	474.993	–0.889	0.374		9.765	0.007	0.023	0.879	10.520	0.001
High-intensity distance (in possession)	2,433.00 ± 548.70	2,190.71 ± 539.06	474.217	–2.582	0.010	H > L	9.695	0.008	0.204	0.652	17.847	<0.001
High-intensity distance 1st half (out of possession)	1,220.61 ± 323.57	1,309.80 ± 319.25	473.582	2.875	0.004	H < L	3.133	0.098	11.240	0.001	3.446	0.064
High-intensity distance 2nd half (out of possession)	1,250.39 ± 319.62	1,311.46 ± 353.58	463.252	1.169	0.243		1.279	0.276	11.525	0.001	6.813	0.009
High-intensity distance (out of possession)	2,467.41 ± 539.01	2,614.02 ± 556.85	474.463	2.414	0.016	H < L	2.552	0.132	16.457	<0.001	7.156	0.008
High-intensity distance 1st half	2,515.59 ± 507.24	2,473.89 ± 527.50	474.757	0.364	0.716		10.368	0.006	7.911	0.005	1.374	0.242
High-intensity distance 2nd half	2,579.41 ± 547.80	2,521.42 ± 563.94	474.375	–0.862	0.389		6.768	0.021	7.336	0.007	0.146	0.702
High-intensity distance	5,060.68 ± 968.04	4,945.17 ± 939.06	474.638	–0.071	0.944		8.233	0.012	4.409	0.036	1.827	0.177
High-intensity average interval (s)	211.13 ± 37.86	215.74 ± 40.40	474.885	–0.094	0.925		8.088	0.013	8.915	0.003	0.878	0.349
Sprint distance 1st half (in possession)	562.91 ± 196.21	531.58 ± 185.54	472.442	–0.243	0.808		10.954	0.005	3.682	0.056	16.546	<0.001
Sprint distance 2nd half (in possession)	579.00 ± 211.13	544.31 ± 183.59	474.699	0.442	0.659		14.293	0.002	1.828	0.177	15.778	<0.001
Sprint distance (in possession)	1,136.93 ± 392.84	1,075.80 ± 312.29	474.948	0.392	0.695		15.508	0.001	0.002	0.963	24.424	<0.001
Sprint distance 1st half (out of possession)	521.83 ± 182.56	540.21 ± 116.70	473.517	1.036	0.301		2.751	0.119	5.606	0.018	1.069	0.302
Sprint distance 2nd half (out of possession)	547.21 ± 173.91	544.80 ± 200.93	460.935	–0.940	0.348		0.710	0.413	14.902	<0.001	6.387	0.012
Sprint distance (out of possession)	1,068.33 ± 289.70	1,079.89 ± 288.04	474.319	–0.318	0.751		1.985	0.180	18.259	<0.001	6.913	0.009
Sprint distance 1st half	1,109.76 ± 294.31	1,092.27 ± 285.51	474.824	0.588	0.557		8.706	0.010	6.891	0.009	4.067	0.044
Sprint distance 2nd half	1,159.39 ± 277.93	1,135.60 ± 392.92	474.378	0.525	0.600		9.032	0.009	0.454	0.501	2.390	0.123
Sprint distance	2,283.41 ± 542.01	2,243.15 ± 589.66	474.679	0.698	0.485		11.283	0.005	2.560	0.110	6.750	0.010

*HPBPT, high percentage ball possession team; LPBPT, low percentage ball possession team.*

Table 3 illustrates the running indicators across playing positions in HPBPT and LPBPT. HPBPT fullbacks covered more high-intensity running (second half and total) and sprint distance (second half and total) than fullbacks in LPBPT (*p* < 0.05). Central defenders of HPBPT covered more high intensity distance (second half and total) and sprint distance (second half and total), as well as less high-intensity average intervals than central defenders in LPBPT (*p* < 0.05). However, wide midfielders of HPBPT covered less high-intensity running (second half, total) and sprint distance (second half and total) and more high-intensity average intervals than wide midfielders in LPBPT (*p* < 0.05). Additionally, attackers of HPBPT covered less high-intensity running (first half, second half, and total) and sprint distance (first half, second half, and total) than their LPBPT counterparts (*p* < 0.05). No differences were observed for central midfielders between HPBPT and LPBPT. As covariates, in general, QO influenced the high speed performance of fullbacks and central backs, and ML had an impact on the high speed running of wide midfielders and attackers.

**TABLE 3:** Running performance in terms of playing position between HPBPT and LPBPT.

	Fullbacks											
Indicators	HPBPT	LPBPT	df	Statistic(t)	*p*-value	*Post hoc* comparison	Team strength		Quality of opposition		Match location	
									
							Statistic(t)	*p*-value	Statistic(t)	*p*-value	Statistic(t)	*p*-value
TD 1st half	4,295.96 ± 1709.10	4,249.11 ± 1783.96	5858.328	–0.569	0.570		1.309	0.268	0.151	0.698	0.228	0.633
TD 2nd half	4,193.27 ± 1520.24	4,163.40 ± 1511.92	5879.893	–0.677	0.498		0.002	0.969	1.024	0.312	1.295	0.255
TD	8,489.64 ± 2020.71	8,418.76 ± 2951.71	5862.368	–0.706	0.480		0.760	0.395	0.861	0.354	0.899	0.343
HID 1st half	245.75 ± 139.60	226.51 ± 131.35	5911.284	–1.357	0.175		0.778	0.390	5.224	0.022	0.518	0.472
HID 2nd half	239.36 ± 124.85	212.28 ± 116.66	5958.618	–3.820	<0.001	H > L	1.226	0.282	23.159	<0.001	1.801	0.180
HID	485.56 ± 222.97	438.22 ± 206.75	5866.452	–3.357	0.001	H > L	0.856	0.367	18.814	<0.001	0.572	0.450
HIAI (s)	190.76 ± 97.04	204.13 ± 99.94	5990.931	0.643	0.520		3.239	0.090	16.944	<0.001	0.210	0.647
SD 1st half	116.12 ± 79.06	105.19 ± 75.33	5949.763	–1.523	0.128		2.158	0.161	4.450	0.035	2.325	0.128
SD 2nd half	113.30 ± 71.72	94.79 ± 67.09	5990.719	–4.380	<0.001	H > L	1.404	0.251	24.619	<0.001	1.587	0.208
SD	228.36 ± 124.67	199.43 ± 115.36	5887.002	–3.755	<0.001	H > L	1.415	0.250	19.153	<0.001	0.017	0.895

**Indicators**	**Central defenders**											
	**HPBPT**	**LPBPT**	**df**	**Statistic(t)**	***p*-value**	***Post hoc* comparison**	**Team strength**		**Quality of opposition**		**Match location**	
									
							**Statistic(t)**	***p*-value**	**Statistic(t)**	***p*-value**	**Statistic(t)**	***p*-value**

TD 1st half	4,233.51 ± 1335.39	3,996.94 ± 1549.03	5857.022	–0.898	0.369		0.051	0.824	0.089	0.765	1.219	0.270
TD 2nd half	4,210.82 ± 1121.39	4,004.86 ± 1259.52	5879.501	–1.560	0.119		0.574	0.460	0.038	0.845	1.474	0.225
TD	8,449.44 ± 2257.38	8,001.46 ± 2580.50	5860.899	–1.306	0.192		0.258	0.618	0.047	0.829	1.857	0.173
HID 1st half	145.98 ± 80.61	127.08 ± 79.91	5913.368	–0.442	0.659		0.376	0.550	0.687	0.407	0.256	0.613
HID 2nd half	160.39 ± 87.84	128.76 ± 86.17	5965.097	–4.205	<0.001	H > L	2.396	0.142	11.335	0.001	0.464	0.496
HID	305.64 ± 136.27	255.84 ± 128.65	5865.038	–2.711	0.007	H > L	0.089	0.770	7.727	0.006	0.621	0.431
HIAI (s)	325.29 ± 223.58	375.09 ± 253.52	6006.222	3.850	<0.001	H<L	0.010	0.921	3.080	0.080	1.478	0.224
SD 1st half	65.34 ± 49.45	53.94 ± 44.20	5954.325	–1.027	0.305		0.776	0.394	2.083	0.149	0.015	0.902
SD 2nd half	71.66 ± 49.67	57.15 ± 47.17	5999.772	–3.258	0.001	H > L	1.485	0.225	7.043	0.008	3.222	0.073
SD	136.23 ± 79.16	110.92 ± 72.96	5886.778	–2.605	0.009	H > L	0.001	0.973	6.900	0.009	1.458	0.228

**Indicators**	**Wide midfielders**											
	**HPBPT**	**LPBPT**	**df**	**Statistic(t)**	***p*-value**	***Post hoc* comparison**	**Team strength**		**Quality of opposition**		**Match location**	
									
							**Statistic(t)**	***p*-value**	**Statistic(t)**	***p*-value**	**Statistic(t)**	***p*-value**

TD 1st half	3,735.75 ± 2353.33	3,751.15 ± 2400.84	5841.098	0.093	0.926		2.588	0.139	0.429	0.513	0.005	0.946
TD 2nd half	3,589.64 ± 1774.07	3,648.59 ± 1819.89	5860.181	1.807	0.071		10.954	0.007	2.963	0.085	0.391	0.532
TD	7,328.80 ± 3489.21	7,398.35 ± 3536.43	5845.657	0.972	0.331		7.513	0.021	2.062	0.151	0.044	0.833
HID 1st half	222.33 ± 169.70	233.57 ± 180.10	5886.592	1.430	0.153		0.007	0.934	0.212	0.645	0.776	0.378
HID 2nd half	216.19 ± 138.00	232.01 ± 143.03	5923.346	3.454	0.001	H < L	3.265	0.097	2.087	0.149	4.267	0.039
HID	436.52 ± 253.41	465.01 ± 263.38	5849.271	3.360	0.001	H < L	1.073	0.321	0.180	0.671	4.162	0.042
HIAI (s)	172.08 ± 104.81	155.23 ± 86.19	5933.065	–2.452	0.014	H > L	2.400	0.143	0.080	0.778	0.097	0.043
SD 1st half	101.65 ± 93.25	107.33 ± 95.04	5919.608	1.065	0.287		0.489	0.497	0.536	0.464	1.825	0.177
SD 2nd half	98.45 ± 78.70	107.12 ± 81.22	5951.721	3.442	0.001	H < L	0.070	0.796	3.359	0.067	4.026	0.045
SD	199.14 ± 140.07	213.38 ± 177.96	5867.654	2.997	0.003	H < L	0.020	0.889	0.353	0.553	6.031	0.014

**Indicators**	**Central midfielders**											
	**HPBPT**	**LPBPT**	**df**	**Statistic(t)**	***p*-value**	***Post hoc* comparison**	**Team strength**		**Quality of opposition**		**Match location**	
									
							**Statistic(t)**	***p*-value**	**Statistic(t)**	***p*-value**	**Statistic(t)**	***p*-value**

TD 1st half	3,821.33 ± 2332.14	3,844.13 ± 2368.59	5840.877	1.616	0.106		0.001	0.982	0.019	0.891	0.022	0.881
TD 2nd half	3,962.65 ± 1680.12	3,892.17 ± 1778.24	5862.348	0.689	0.491		0.016	0.901	0.085	0.771	0.253	0.615
TD	7787.61 ± 3532.28	7704.72 ± 3761.78	5845.382	1.000	0.317		0.000	0.998	0.050	0.823	0.002	0.963
HID 1st half	162.86 ± 129.26	156.58 ± 128.17	5894.710	0.652	0.515		0.643	0.436	3.867	0.049	0.698	0.404
HID 2nd half	184.70 ± 113.48	178.28 ± 113.05	5943.696	0.239	0.811		2.311	0.149	0.198	0.656	0.159	0.690
HID	346.33 ± 203.98	335.98 ± 211.96	5850.742	0.952	0.341		1.324	0.267	0.907	0.341	0.814	0.367
HIAI (s)	223.78 ± 123.96	236.94 ± 180.09	5978.321	0.752	0.452		5.316	0.038	2.090	0.148	1.768	0.184
SD 1st half	64.36 ± 65.51	61.68 ± 62.59	5934.725	0.738	0.461		0.492	0.494	3.896	0.049	1.903	0.168
SD 2nd half	78.28 ± 65.29	73.60 ± 63.07	5977.322	–0.058	0.954		2.859	0.112	0.102	0.749	2.147	0.143
SD	141.36 ± 107.07	134.21 ± 104.43	5871.130	0.731	0.465		1.533	0.234	0.913	0.339	3.466	0.063

**Indicators**	**Attackers**											
	**HPBPT**	**LPBPT**	**df**	**Statistic(t)**	***p*-value**	***Post hoc* comparison**	**Team strength**		**Quality of opposition**		**Match location**	
									
							**Statistic(t)**	***p*-value**	**Statistic(t)**	***p*-value**	**Statistic(t)**	***p*-value**

TD 1st half	3,615.17 ± 2213.77	3,832.87 ± 2137.69	5882.639	1.253	0.210		0.504	0.490	0.524	0.470	0.149	0.700
TD 2nd half	3,833.70 ± 1534.15	3,930.78 ± 1498.40	5905.820	–0.583	0.560		0.946	0.347	4.888	0.027	0.854	0.356
TD	7,449.91 ± 3300.06	7,762.28 ± 3236.64	5885.866	–1.077	0.282		0.159	0.697	0.441	0.507	0.315	0.575
HID 1st half	209.60 ± 153.66	231.91 ± 159.01	5937.756	2.535	0.011	H < L	0.194	0.667	1.519	0.218	3.265	0.017
HID 2nd half	208.41 ± 115.56	231.50 ± 121.61	5987.020	2.838	0.005	H < L	10.004	0.006	0.045	0.832	5.126	0.024
HID	418.14 ± 221.64	463.23 ± 237.02	5887.470	3.393	0.001	H < L	0.784	0.392	0.335	0.563	5.971	0.015
HIAI (s)	180.64 ± 89.55	178.05 ± 107.59	6025.176	–0.932	0.351		3.231	0.074	0.831	0.362	6.989	0.008
SD 1st half	96.08 ± 81.53	113.09 ± 93.63	5976.536	3.059	0.002	H < L	0.018	0.896	0.434	0.510	0.730	0.393
SD 2nd half	95.65 ± 68.89	112.83 ± 76.81	6020.422	3.526	<0.001	H<L	10.802	0.005	2.117	0.146	1.163	0.281
SD	191.76 ± 122.46	225.43 ± 142.01	5909.357	4.199	<0.001	H<L	2.160	0.165	0.511	0.475	1.371	0.242

*HPBPT, high percentage ball possession team; LPBPT, low percentage ball possession team; TD, total distance; HID, high intensity distance; HIAI, high intensity average interval; SD, sprint distance.*

### Technical-Tactical Performance

Table 4 shows the technical and tactical performance indicators for HPBPT and LPBPT. HPBPT performed better in their offensive indicators than LPBPT. These indicators included shots, total passes, successful passes, pass success rates, forward passes, success rates of forward passes, corners, crosses, possession in opponent’s half, opponent 35 m entry, opponent penalty area entry, and success rate of aerial challenges (*p* < 0.05). However, LPBPT played more ground challenges than HPBPT (*p* = 0.002). As covariates, TS, QO, and ML also had a certain impact on the teams’ technical performance.

**TABLE 4:** Difference of technical performance between HPBPT and LPBPT.

Indicators	HPBPT	LPBPT	df	Statistic(t)	*p*-value	*Post hoc* comparison	Team strength		Quality of opposition		Match location	
									
							Statistic(t)	*p*-value	Statistic(t)	*p*-value	Statistic(t)	*p*-value
Shots	14.63 ± 4.89	11.44 ± 3.89	467.787	–5.583	<0.001	H > L	9.774	0.007	2.116	0.146	35.619	<0.001
Shots on target	5.39 ± 2.84	4.43 ± 2.37	462.361	–1.102	0.271		16.710	0.001	9.121	0.003	31.610	<0.001
Total passes	430.48 ± 87.73	306.18 ± 67.68	470.328	–15.109	<0.001	H > L	3.358	0.088	0.266	0.606	3.862	0.050
Successful passes	353.34 ± 86.80	232.00 ± 67.72	469.817	–14.781	<0.001	H > L	3.484	0.083	0.258	0.611	3.872	0.050
Pass success rate (%)	81.81 ± 4.34	75.02 ± 6.10	471.786	–11.617	<0.001	H > L	4.034	0.064	0.448	0.504	1.200	0.274
Forward passes	138.12 ± 23.13	112.67 ± 19.04	473.757	–10.991	<0.001	H > L	2.780	0.117	0.990	0.320	6.007	0.015
Success rate of forward pass (%)	65.74 ± 7.00	57.83 ± 7.77	474.569	–9.694	<0.001	H > L	10.334	0.006	2.089	0.149	2.843	0.092
Crosses	19.30 ± 7.15	13.31 ± 4.88	474.371	–9.854	<0.001	H > L	0.011	0.919	0.000	0.994	6.171	0.013
Corners	5.81 ± 2.85	4.23 ± 2.07	467.787	–5.583	<0.001	H > L	5.869	0.016	0.717	0.398	11.137	0.001
Possession in opponent’s half (%)	47.84 ± 6.50	41.76 ± 5.93	474.000	–9.068	<0.001	H > L	2.245	0.156	2.262	0.133	10.141	0.002
Opponent 35 m entry	52.20 ± 1.66	36.66 ± 8.50	433.540	–14.635	<0.001	H > L	12.566	0.003	0.049	0.825	6.616	0.010
Opponent penalty area entry	8.96 ± 4.07	5.83 ± 2.82	461.462	–7.306	<0.001	H > L	12.573	0.003	8.052	0.005	17.740	<0.001
Challenges won (%)	50.15 ± 6.68	49.76 ± 7.38	414.924	0.728	0.467		6.604	0.021	8.742	0.003	0.628	0.429
Aerial challenges	31.30 ± 9.10	31.02 ± 9.01	474.967	–1.658	0.098		1.478	0.244	2.802	0.095	0.209	0.648
Number of successful aerial challenges	15.38 ± 5.33	14.88 ± 5.57	474.330	–0.678	0.498		0.115	0.740	13.827	<0.001	6.748	0.010
Success rate of aerial challenges (%)	47.79 ± 13.70	47.43 ± 11.95	458.438	2.126	0.034	H > L	20.747	0.000	12.990	<0.001	6.885	0.009
Ground challenges	58.64 ± 13.22	61.63 ± 14.20	470.093	3.054	0.002	H < L	8.061	0.013	4.233	0.040	0.010	0.920
Number of successful ground challenges	30.87 ± 7.75	32.01 ± 8.81	464.358	1.876	0.061		7.038	0.019	4.182	0.041	0.352	0.553
Success rate of ground challenges (%)	53.25 ± 8.72	52.50 ± 8.52	355.715	–1.104	0.271		0.460	0.508	0.008	0.930	0.679	0.410
Fouls	15.09 ± 4.27	15.20 ± 4.21	474.693	–0.232	0.816		0.030	0.864	0.935	0.334	0.976	0.324
Offsides	1.90 ± 7.737	1.96 ± 1.61	468.967	0.879	0.380		0.614	0.446	0.457	0.499	0.036	0.850
Yellow cards	1.96 ± 1.22	2.09 ± 1.35	466.038	–0.170	0.865		1.589	0.227	0.154	0.695	3.882	0.049
Red cards	0.10 ± 0.33	0.12 ± 0.34	352.086	0.590	0.556		0.340	0.560	1.167	0.281	0.728	0.394

*HPBPT, high percentage ball possession team; LPBPT, low percentage ball possession team.*

The technical and tactical performance indicators across playing positions in HPBPT and LPBPT are illustrated in Table 5. Technical indicators such as total passes, successful passes, pass success rates, forward passes, success rates of forward passes, crosses and red cards were higher among fullbacks in HPBPT than in LPBPT (*p* < 0.05). LPBPT fullbacks carried out more ground challenges than their HPBPT counterparts (*p* < 0.05). HPBPT central defenders recorded more total passes, successful passes, pass success rates, forward passes, success rates of forward passes, aerial challenges and number of successful aerial challenges than their LPBPT counterparts (*p* < 0.05). HPBPT wide midfielders also recorded more total passes, successful passes, pass success rates, forward passes, and crosses than their LPBPT counterparts (*p* < 0.05). However, there were slightly more ground challenges for wide midfielders from LPBPT than those from HPBPT (*p* = 0.001). HPBPT central midfielders also recorded more shots, shot success rates, total passes, successful passes, pass success rates, forward passes, forward pass success rates, crosses, and cross success rates than players in the same position from LPBPT (*p* < 0.05). However, there were slightly higher success rates of aerial challenges and ground challenges for central midfielders from LPBPT than those from HPBPT (*p* < 0.05). Compared to LPBPT, attackers from HPBPT recorded more shots, successful passes, pass success rates and crosses (*p* < 0.05) but had poorer shots success rates (*p* = 0.011) and aerial challenges (*p* = 0.028). As covariates, TS mainly affected AC and the AC success rate in fullbacks; GC success rate in central backs; shots success rate and AC success rate in wide midfielders; AC success rate and yellow cards in central midfielders; and F-passes success rate and shots success rate in forward. QO had impacts on AC success rate in fullbacks; on AC, AC success and its rate in central defenders; on F-passes success rate in wide midfielders; on F-passes and yellow cards in central midfielders; and on pass success rate in forward. ML mostly impacted pass related variables in fullbacks; passes and their success rate, GC and fouls in central backs; crosses in wide midfielders; shots and crosses in central midfielders; and shots, shots success rate, passes, AC and yellow cards in forward.

**TABLE 5:** Technical and tactical performance in terms of playing position between HPBPT and LPBPT.

Indicators	Fullbacks											
	HPBPT	LPBPT	df	Statistic(t)	*p*-value	*Post hoc* comparison	Team strength		Quality of opposition		Match location	
									
							Statistic(t)	*p*-value	Statistic(t)	*p*-value	Statistic(t)	*p*-value
Shots	0.39 ± 0.68	0.27 ± 0.57	5923.212	–1.524	0.127		0.032	0.861	0.248	0.619	1.393	0.238
Shots SCR (%)	6.61 ± 23.41	5.46 ± 21.62	6149.893	–0.109	0.913		1.696	0.179	3.022	0.082	2.687	0.101
Passes	35.48 ± 17.26	23.83 ± 12.65	5848.482	–12.899	<0.001	H > L	0.067	0.798	0.378	0.539	4.166	0.042
Passes SC	29.38 ± 15.35	18.16 ± 10.63	5907.508	–12.446	<0.001	H > L	0.061	0.807	0.581	0.446	5.811	0.016
Passes SCR (%)	81.15 ± 12.96	73.30 ± 18.70	6105.993	–6.109	<0.001	H > L	1.289	0.280	2.132	0.145	0.340	0.560
F-Passes	13.66 ± 7.19	10.88 ± 6.10	5895.695	–8.766	<0.001	H > L	1.943	0.173	0.043	0.836	4.866	0.028
F-Passes SCR (%)	65.17 ± 21.21	57.62 ± 23.75	6083.727	–4.100	<0.001	H > L	1.763	0.205	1.545	0.214	2.111	0.147
Crosses	2.60 ± 2.58	1.97 ± 2.14	5920.627	–5.957	<0.001	H > L	0.185	0.672	0.470	0.493	2.204	0.138
Crosses SCR (%)	17.48 ± 25.93	17.10 ± 29.61	5976.526	0.041	0.968		1.345	0.265	0.764	0.382	4.649	0.031
AC	2.02 ± 1.78	1.77 ± 1.69	5928.869	–1.485	0.138		2.973	0.103	2.975	0.085	0.281	0.596
AC SC	1.42 ± 1.28	1.28 ± 1.22	5969.131	–0.743	0.457		6.905	0.010	7.407	0.007	0.056	0.812
AC SCR (%)	55.63 ± 38.55	54.12 ± 39.13	6149.742	–0.679	0.497		5.267	0.025	0.970	0.325	1.149	0.284
GC	4.63 ± 2.97	4.96 ± 3.16	6019.262	2.304	0.021	H < L	0.277	0.606	2.354	0.125	0.929	0.335
GC SC	2.87 ± 1.88	2.93 ± 1.97	6036.032	1.275	0.202		0.417	0.527	0.830	0.363	2.604	0.107
GC SCR (%)	59.81 ± 27.88	56.57 ± 27.11	6094.258	–1.599	0.110		0.137	0.713	0.273	0.601	2.802	0.094
Fouls	1.22 ± 1.18	1.20 ± 1.24	6057.737	0.242	0.809		0.763	0.397	0.598	0.439	0.704	0.402
Y-cards	0.19 ± 0.39	0.17 ± 0.38	5789.599	–1.418	0.156		0.057	0.815	1.594	0.207	1.249	0.264
R- cards	0.02 ± 0.15	0.01 ± 0.09	5426.045	–2.353	0.019	H > L	0.026	0.873	1.685	0.195	2.067	0.151

**Indicators**	**Central defenders**											
	**HPBPT**	**LPBPT**	**df**	**Statistic(t)**	***p*-value**	***Post hoc* comparison**	**Team strength**		**Quality of opposition**		**Match location**	
									
							**Statistic(t)**	***p*-value**	**Statistic(t)**	***p*-value**	**Statistic(t)**	***p*-value**

Shots	0.40 ± 0.76	0.35 ± 0.67	5920.703	–0.001	0.999		0.400	0.529	0.556	0.456	4.323	0.038
Shots SCR (%)	10.37 ± 29.17	9.24 ± 27.33	6146.304	0.144	0.885		0.221	0.645	1.316	0.252	1.489	0.223
Passes	35.97 ± 15.90	21.63 ± 11.81	5845.325	–15.867	<0.001	H > L	0.515	0.484	1.784	0.182	5.862	0.016
Passes SC	31.26 ± 14.79	17.28 ± 10.43	5907.539	–15.685	<0.001	H > L	0.222	0.645	2.553	0.110	7.782	0.005
Passes SCR (%)	84.33 ± 14.68	76.34 ± 18.75	6127.892	–6.138	<0.001	H > L	1.802	0.201	2.984	0.084	1.609	0.205
F-Passes	13.78 ± 7.29	9.32 ± 5.38	5894.521	–12.338	<0.001	H > L	0.268	0.613	0.436	0.509	3.610	0.058
F-Passes SCR (%)	71.03 ± 20.07	59.48 ± 24.94	6105.942	–6.177	<0.001	H > L	0.282	0.604	0.602	0.438	2.377	0.123
Crosses	0.27 ± 0.84	0.13 ± 0.46	5919.354	–0.606	0.545		0.254	0.622	1.231	0.267	0.062	0.803
Crosses SCR (%)	4.00 ± 18.58	2.39 ± 14.65	6005.693	–0.450	0.653		0.084	0.777	0.186	0.666	0.028	0.868
AC	3.60 ± 2.81	3.04 ± 2.43	5925.154	–3.234	0.001	H > L	1.688	0.214	8.790	0.003	0.010	0.922
AC SC	2.58 ± 2.02	2.14 ± 1.76	5971.597	–4.295	<0.001	H > L	2.243	0.136	13.929	<0.001	0.497	0.481
AC SCR (%)	65.29 ± 30.93	62.89 ± 33.55	6145.471	–0.759	0.448		0.121	0.729	6.062	0.014	3.498	0.062
GC	3.96 ± 2.60	4.02 ± 2.73	6032.545	0.731	0.465		0.128	0.726	4.462	0.035	3.897	0.049
GC SC	2.56 ± 1.87	2.63 ± 1.85	6061.114	0.567	0.571		0.039	0.847	1.886	0.170	1.914	0.167
GC SCR (%)	60.80 ± 28.64	62.27 ± 28.56	6051.195	0.550	0.582		4.678	0.034	0.421	0.517	0.139	0.710
Fouls	1.19 ± 1.13	1.03 ± 1.08	6090.745	–1.673	0.094		0.300	0.593	0.478	0.490	10.217	0.001
Y-cards	0.18 ± 0.39	0.19 ± 0.41	5738.730	0.155	0.877		0.012	0.915	0.927	0.336	0.045	0.832
R- cards	0.01 ± 0.12	0.01 ± 0.12	5368.323	–0.272	0.786		0.626	0.441	0.525	0.469	3.318	0.069

**Indicators**	**Wide Midfielders**											
	**HPBPT**	**LPBPT**	**df**	**Statistic(t)**	***p*-value**	***Post hoc* comparison**	**Team strength**		**Quality of opposition**		**Match location**	
									
							**Statistic(t)**	***p*-value**	**Statistic(t)**	***p*-value**	**Statistic(t)**	***p*-value**

Shots	1.28 ± 1.61	1.04 ± 1.37	5879.269	–1.693	0.090		0.736	0.405	2.273	0.132	3.463	0.063
Shots SCR (%)	19.85 ± 33.28	20.29 ± 35.22	6140.035	1.667	0.096		3.866	0.050	2.803	0.094	0.001	0.980
Passes	27.27 ± 19.96	20.59 ± 13.79	5836.920	–6.615	<0.001	H > L	2.354	0.183	2.452	0.118	0.515	0.698
Passes SC	21.81 ± 17.07	15.68 ± 13.19	5886.590	–6.149	<0.001	H > L	1.373	0.279	1.122	0.290	0.390	0.532
Passes SCR (%)	77.10 ± 17.57	71.22 ± 19.48	6028.133	–5.257	<0.001	H > L	0.041	0.840	3.116	0.078	0.850	0.357
F-Passes	7.32 ± 6.73	6.27 ± 5.03	5879.450	–2.262	0.024	H > L	3.117	0.114	0.067	0.795	0.860	0.354
F-Passes SCR (%)	51.28 ± 30.92	49.09 ± 29.81	6007.600	–0.733	0.464		0.010	0.921	5.510	0.019	0.010	0.921
Crosses	3.26 ± 3.40	2.29 ± 2.61	5903.896	–9.390	<0.001	H > L	2.031	0.180	1.231	0.267	5.716	0.017
Crosses SCR (%)	19.02 ± 26.72	16.66 ± 27.91	5788.888	–1.285	0.199		0.011	0.918	0.989	0.320	0.396	0.529
AC	1.62 ± 1.72	1.61 ± 1.72	5870.013	-0.943	0.346		0.364	0.547	0.586	0.444	0.148	0.701
AC SC	0.86 ± 1.01	0.82 ± 1.26	5908.321	0.129	0.898		0.763	0.383	0.804	0.370	1.185	0.277
AC SCR (%)	36.56 ± 38.28	34.96 ± 37.96	6140.875	0.449	0.654		6.630	0.022	2.017	0.156	2.209	0.137
GC	4.38 ± 3.25	4.87 ± 3.48	5964.655	3.368	0.001	H < L	0.000	0.984	0.349	0.555	0.153	0.696
GC SC	2.41 ± 1.88	2.44 ± 1.95	5901.868	1.035	0.301		0.069	0.793	0.206	0.650	0.043	0.836
GC SCR (%)	50.46 ± 28.88	46.44 ± 28.67	6143.033	–1.801	0.072		0.908	0.354	0.104	0.748	1.966	0.161
Fouls	1.02 ± 1.10	1.11 ± 1.24	5902.485	0.870	0.384		0.269	0.604	3.594	0.058	0.886	0.347
Y-cards	0.10 ± 0.32	0.09 ± 0.29	5651.784	–1.205	0.228		0.539	0.474	3.416	0.065	1.636	0.201
R- cards	0.00 ± 0.05	0.01 ± 0.08	5140.819	0.693	0.488		1.542	0.214	0.721	0.396	3.219	0.073

**Indicators**	**Central midfielders**											
	**HPBPT**	**LPBPT**	**df**	**Statistic(t)**	***p*-value**	***Post hoc* comparison**	**Team strength**		**Quality of opposition**		**Match location**	
									
							**Statistic(t)**	***p*-value**	***p*-value**	**Statistic(t)**	**Statistic(t)**	***p*-value**

Shots	1.26 ± 1.64	0.87 ± 1.25	5916.186	–3.777	<0.001	H > L	1.243	0.266	0.448	0.503	9.410	0.002
Shots SCR (%)	18.66 ± 32.85	13.67 ± 30.01	6149.030	–2.138	0.033	H > L	0.854	0.357	0.075	0.785	1.771	0.183
Passes	40.21 ± 25.33	27.87 ± 17.60	5833.985	–14.039	<0.001	H > L	0.055	0.818	2.225	0.136	2.688	0.101
Passes SC	33.84 ± 22.54	21.89 ± 14.80	5892.556	–13.421	<0.001	H > L	0.154	0.700	0.229	0.632	0.740	0.390
Passes SCR (%)	80.34 ± 17.64	74.30 ± 20.08	6100.422	–5.652	<0.001	H > L	0.058	0.812	0.192	0.661	0.261	0.610
F-Passes	11.51 ± 8.58	9.28 ± 6.49	5880.405	–6.042	<0.001	H > L	0.241	0.632	3.992	0.046	2.810	0.094
F-Passes SCR (%)	57.97 ± 27.47	51.70 ± 27.59	6077.987	–3.661	<0.001	H > L	0.191	0.670	0.284	0.594	2.158	0.142
Crosses	1.94 ± 3.00	1.38 ± 2.37	5907.420	–5.850	<0.001	H > L	0.066	0.800	0.352	0.553	5.087	0.024
Crosses SCR (%)	14.17 ± 27.11	10.41 ± 23.69	5920.226	–2.536	0.011	H > L	0.167	0.688	1.153	0.283	1.660	0.198
AC	1.87 ± 2.03	1.76 ± 1.84	5923.947	–2.223	0.026	H > L	0.730	0.394	0.776	0.379	0.021	0.886
AC SC	1.13 ± 1.36	1.12 ± 1.22	5963.700	–0.244	0.807		0.061	0.805	1.816	0.178	1.767	0.184
AC SCR (%)	42.82 ± 39.59	46.01 ± 39.12	6148.589	2.265	0.024	H < L	4.944	0.027	0.454	0.500	0.012	0.914
GC	5.22 ± 3.54	5.28 ± 3.87	6009.378	2.087	0.037	H < L	0.809	0.385	1.831	0.176	0.035	0.853
GC SC	2.82 ± 2.07	2.92 ± 2.22	6036.885	1.811	0.070		3.020	0.083	2.498	0.114	0.481	0.488
GC SCR (%)	50.26 ± 26.63	50.49 ± 27.65	6069.292	–0.311	0.756		1.812	0.180	0.361	0.548	0.623	0.430
Fouls	1.23 ± 1.26	1.29 ± 1.29	6052.825	0.427	0.669		0.482	0.488	0.300	0.584	1.786	0.182
Y-cards	0.15 ± 0.36	0.17 ± 0.38	5649.484	0.559	0.576		5.980	0.016	4.421	0.036	0.242	0.623
R- cards	0.00 ± 0.07	0.01 ± 0.10	5146.313	1.149	0.251		0.158	0.692	1.839	0.175	1.167	0.280

**Indicators**	**Attackers**											
	**HPBPT**	**LPBPT**	**df**	**Statistic(t)**	***p*-value**	***Post hoc* comparison**	**Team strength**		**Quality of opposition**		**Match location**	
									
							**Statistic(t)**	***p*-value**	**Statistic(t)**	***p*-value**	**Statistic(t)**	***p*-value**

Shots	2.47 ± 2.13	2.16 ± 1.83	5902.444	–3.584	0.000	H > L	2.258	0.135	0.921	0.337	11.905	0.001
Shots SCR (%)	32.92 ± 34.37	37.41 ± 38.13	6125.333	2.552	0.011	H < L	6.216	0.014	0.015	0.902	8.722	0.003
Passes	20.40 ± 13.62	19.49 ± 11.83	5866.585	–1.230	0.219		2.285	0.162	4.040	0.045	4.292	0.039
Passes SC	14.62 ± 10.89	13.05 ± 8.89	5928.887	–2.467	0.014	H > L	2.267	0.134	1.526	0.217	0.040	0.841
Passes SCR (%)	68.54 ± 20.49	63.05 ± 21.39	6135.492	–3.377	0.001	H > L	1.937	0.192	4.607	0.032	2.843	0.092
F-Passes	4.65 ± 3.83	5.23 ± 4.16	5916.970	0.666	0.506		1.362	0.244	1.500	0.221	2.904	0.089
F-Passes SCR (%)	40.94 ± 32.91	39.98 ± 29.87	6109.674	–0.607	0.544		9.679	0.003	1.444	0.230	0.579	0.447
Crosses	1.45 ± 2.18	1.26 ± 1.82	5937.734	–1.997	0.046	H > L	2.534	0.145	1.972	0.161	1.864	0.173
Crosses SCR (%)	11.68 ± 25.94	11.40 ± 25.87	5806.813	–0.117	0.907		0.977	0.341	3.261	0.071	1.225	0.269
AC	4.10 ± 3.76	4.85 ± 4.78	5876.236	2.195	0.028	H < L	0.685	0.422	0.262	0.609	4.219	0.040
AC SC	2.00 ± 2.07	2.39 ± 2.68	5939.880	1.747	0.081		0.219	0.641	0.218	0.640	0.822	0.365
AC SCR (%)	38.02 ± 29.37	35.60 ± 28.55	6122.843	–0.218	0.827		1.645	0.220	0.178	0.673	1.554	0.213
GC	4.27 ± 3.04	4.59 ± 3.13	6042.486	1.636	0.102		2.036	0.179	1.633	0.202	0.514	0.474
GC SC	2.18 ± 1.75	2.34 ± 1.85	5986.126	1.177	0.239		2.004	0.159	1.984	0.159	0.002	0.968
GC SCR (%)	45.93 ± 28.84	47.31 ± 28.38	5953.512	0.770	0.441		0.199	0.663	1.518	0.218	0.120	0.729
Fouls	1.26 ± 1.34	1.25 ± 1.30	6002.938	–0.221	0.825		0.057	0.811	0.242	0.623	0.595	0.441
Y-cards	0.13 ± 0.35	0.13 ± 0.34	5483.824	–0.361	0.718		1.377	0.257	0.014	0.907	4.324	0.038
R- cards	0.01 ± 0.08	0.01 ± 0.08	5021.689	–0.152	0.879		0.415	0.521	0.724	0.395	0.005	0.943

*AC, aerial challenges; F, forward; GC, ground challenges; P, passes; R-card, red card; SC, success; SCR, success rate; Y-cards, Yellow cards.*

## Discussion

The aim of this study was to analyze physical fitness and technical-tactical performance under high and low ball possession (BP) in different playing positions in the CSL, while contextual variables including team strength (TS), quality of opposition (QO), and match location (ML) were also considered as covariates. To our knowledge, this is the first study that analyses activities in terms of ball possession and playing positions in the CSL. The main findings were as follows: (1) high-intensity running with and without ball is the major difference between high possession and low possession teams; positionally, central defenders and fullbacks in high possession teams covered more high-intensity and sprint running distance, while wide midfielders and forward from low possession teams covered more high-intensity and sprint running distance. (2) Teams with high possession and low possession exhibited differences in attacking organization variables, including quantity and quality. Moreover, high possession teams may be made up of players with a higher technical and tactical performance.

Running performance is widely studied by researchers. Compared to total running distance, which alone is not a key indicator for achieving success (Hoppe et al., 2015; Jiang et al., 2018), high-intensity running and sprinting are especially important (Mohr et al., 2003), since they are directly associated with match outcome (Stolen et al., 2005; Faude et al., 2012; Wu and Zhang, 2017) and team ranking at the end of the season (Di Salvo et al., 2009; Rampinini et al., 2009; Hoppe et al., 2015). Previous studies on the CSL also suggested that high-intensity running plays a more critical role than total running distance (Wu and Zhang, 2017), similar to the present study. Thus, our results for high-intensity running distance are similar to prior studies conducted on the CSL (Wu and Zhang, 2017; Yang et al., 2018). In our study, high possession teams and low possession teams did not show significant differences in total high-intensity running distance, but there were differences found when teams had or did not have ball possession. High possession teams recorded more high-intensity running when they were in possession of the ball, whereas low possession teams recorded more high-intensity running when they were not in possession. This finding is in line with Bradley et al.(2013b, p. 1266), who also found “more distance covered by players in high-intensity running with than without the ball in HPBPT compared to LPBPT” (Bradley et al., 2013b).

Given that high possession teams are strongly associated with success (Lago-Peñas and Dellal, 2010), in the FA Premier League, Di Salvo et al. (2009) found that the five best teams also covered more high-intensity distance when they were in possession while middle and bottom teams (15 teams) ran more intensively than the top 5 teams when they were not in possession. Similar results were also reported in the German Bundesliga by Hoppe et al. (2015), where high-intensity distance with ball possession predicted the majority (60%) of the final rankings. This can be explained by the theory that “it is not match running performance alone that is important for achieving success, but rather its relation to technical/tactical skills with regard to ball possession” (Hoppe et al., 2015, p. 565). Maintaining ball possession through a successful pass is critical, which is probably why high ball possession teams covered less high-intensity distance than low ball possession teams when they were in possession, by using perfect techniques/tactics and keeping the opposition running for ball recovery (da Mota et al., 2016). This is consistent with research findings in the English Premier League (Bradley et al., 2013a).

Despite the importance of running intensity, some studies have stated that technical indicators determine team success more accurately than physical indicators (Di Salvo et al., 2009; Carling, 2013). Indeed, current findings show that high ball possession teams have a higher technical and tactical performance than low possession teams. These findings are supported by previous research studies (Bradley et al., 2013b). Both the quantity and quality of shots and attack organization-related indicators (e.g., forward pass success, pass success rate) are all positively related to high ball possession. High ball possession teams recorded more possession in the opponent’s half, final 1/3 entries, and penalty area entries, which were linked with high-intensity actions (Kai et al., 2018) and shooting opportunities (Lago, 2009; Tenga and Sigmundstad, 2011; Bradley et al., 2014). These important technical and tactical indicators are also key indicators of successful teams in the CSL (Yang et al., 2018) and can reveal the players’ good skills in high ball possession teams.

Using a wide range of CSL player samples, the current results showed that the players’ running performances were different in different playing positions. Physically, fullbacks and center backs from high ball possession teams covered more high-intensity distance and had fewer high-intensity average intervals, while wide midfielders and attackers from low ball possession teams had certain higher high-intensity indicators than their high ball possession team counterparts. Center midfielders were similar in running performance in both high/low possession teams. These results are interesting because the running performance of fullbacks from high ball possession teams or successful teams is already greater than that of wide midfielders. Prior studies found strong correlations between playing position and player running performance, especially in high-intensity running (Bloomfield et al., 2007; Di Salvo et al., 2007, 2009; Bradley et al., 2009; Lago-Peñas et al., 2009; Mallo et al., 2015), where midfielders recorded more total distance and high-intensity running than any other position (Di Salvo et al., 2007, 2009; Bradley et al., 2009; Lago-Peñas et al., 2009; Rampinini et al., 2009).

These published data were reported approximately 10 years ago and during this decade world football has evolved rapidly. For example, studies on increased high-intensity running and sprinting distance (Barnes et al., 2014; Zhou et al., 2020). Bradley et al. (2013b) found similar results, where fullbacks from high ball possession teams performed more high-intensity running and sprinting. Furthermore, there were some recent findings showing that fullbacks covered greater high-intensity and sprint distance than wide midfielders (Vardakis et al., 2019; Aquino et al., 2020). This might be due to playing formation (playing style culture), but it could be due to the football development trend, which is “total possession play.” Typical examples are Spain (2008–2012) and Germany (2014–2018); these players push forward (very hard) when attacking and start to press opponent players immediately after losing possession. Since this playing style became popular around the football world, strong teams tended to adopt this style first, and team formation became increasingly narrow and principally moved more as a whole than ever before. On the one hand, when attacking, three lines of players move together deep into the opponents’ half. In this case, center backs and fullbacks cover a greater distance than ever before, and fullbacks (side backs) play the role of early wide midfielders who are already positioned inside, leaving a passage at the wing to fullbacks. On the other hand, when defending, fullbacks and center backs must run intensively or even sprint to mark opponent players or chase the ball and opponent attackers until the ball is intercepted, resulting in a fast counterattack. This could explain why defenders cover more distance with a different kind of running because high ball possession teams are capable of gaining more entry into their opponents’ half, attacking 1/3 zones, and the penalty area, which are relatively far from their own goal. Consequently, side backs need to run more at high intensity and sprint. Meanwhile, low ball possession teams have to choose a counterattack strategy when facing quality opponents because low possession teams have fewer chances of achieving penetrative passes, so they have to exploit any weaknesses in their strong opponents’ defense and effectively take advantage of an imbalanced defense (Tenga et al., 2010b; Lago-Ballesteros et al., 2012). In this case, forward and side midfielders from low ball possession teams will perform many high-intensity and sprint runs. In addition, since many counterattacks are frequently taken through the side area, side midfielders from low ball possession teams need to both attack and defend (overlapping run) so they have fewer high-intensity average intervals.

Technically, players in different positions from high ball possession teams, especially defenders and midfielders, record a higher technical performance in most indicators in offense (organizing) and shots. These findings are consistent with previous studies regarding high and low possession (Bradley et al., 2013b; da Mota et al., 2016). Moreover, it could be suggested that teams with the skills to sustain possession (or long passing sequences) have a better chance of creating shooting opportunities and thus scoring goals (Hughes and Franks, 2005). That is probably the reason why high ball possession teams are usually strong teams. Strong teams have good players who are perfect at finishing techniques in spite of intense competition and small spaces, and good players are good physically (Mohr et al., 2003) so that they can maintain their playing level and playing style. Bradley et al. (2013b) pointed out that high-intensity running with ball possession and passing ability are very important for a high percentage ball possession strategy, which is line with the results of this study. Good ball passing and control skills are critical for every playing position, although demand varies for different positions. In modern football, defenders have begun to play partial roles as midfielders, midfielders have some overlap with forward, and forward tend to form the first line of starting defense. These changes require players not only to be able to play their own position but also other positions, all of which require good techniques. In this research study, fullbacks from high ball possession teams passed even more than wide midfielders from low ball possession teams. These findings are in line with the UEFA Champions league (Yi et al., 2018), where defenders have become launching points that are greatly involved in attacking (Bush et al., 2015a; Liu et al., 2016) because of the abovementioned “football trend” in recent decades, indicating that defenders today already play in a more important position than ever before.

In this study, TS, QO, and ML had a certain influence along with ball possession on the performance indicators, but did not interfere greatly with the possession effect. The high possession teams had their own advantages and this playing style represents a football trend which needs to be studied and understood. The limitation for this study was that we did not consider possession in the opponents’ half and attacking 1/3 zones as independent variables. Further research studies are warranted and should include these two factors, which could help us to more precisely understand the influence of possession because always passing in one’s own half has not been shown to risk opponent goals and win the match. Additionally, future studies should include more samples (other countries), categories (different competitive levels), genders (women), and ages (youth players).

## Conclusion

Our main findings demonstrated that ball possession influenced team performance both physically and technically, primarily in high-intensity running, sprinting in possession, and high-intensity running out of possession, as well as attack-related indicators such as shots, passing, and entry into opposition areas. Defenders from high ball possession teams engaged in more high-intensity and sprint running, whereas wide midfielders and attackers from low ball possession teams engaged in more high-intensity running and sprinting. Meanwhile, players from high ball possession teams were strong in technical indicators, especially in attacking organization. These results may help coaches to understand current football development trends and develop suitable training plans and tests for elite football players. This study could also be a guide for the development of longitudinal youth football training plans.

## Data Availability Statement

The original contributions presented in the study are included in the article/supplementary material, further inquiries can be directed to the corresponding author/s.

## Author Contributions

TL conceptualized the study and wrote the original draft preparation. TL and LY contributed to the methodology. LY and HC contributed to data collection and visualization. TL, LY, HC, and AG-d-A reviewed and edited the manuscript. AG-d-A helped to improve this work. All authors contributed to the article and approved the submitted version.

## Conflict of Interest

The authors declare that the research was conducted in the absence of any commercial or financial relationships that could be construed as a potential conflict of interest.

## Publisher’s Note

All claims expressed in this article are solely those of the authors and do not necessarily represent those of their affiliated organizations, or those of the publisher, the editors and the reviewers. Any product that may be evaluated in this article, or claim that may be made by its manufacturer, is not guaranteed or endorsed by the publisher.
